# Increase in Female Liver Cancer in The Gambia, West Africa: Evidence from 19 Years of Population-Based Cancer Registration (1988–2006)

**DOI:** 10.1371/journal.pone.0018415

**Published:** 2011-04-07

**Authors:** Dominique Sighoko, Maria Paula Curado, Denis Bourgeois, Maimuna Mendy, Pierre Hainaut, Ebrima Bah

**Affiliations:** 1 International Agency for Research on Cancer, Lyon, France; 2 Faculté d'Odontologie, Méthodes et Algorithmes pour l'Aide à la Décision, Lyon, France; 3 Medical Research Council, Fajara, The Gambia; 4 Gambia Hepatitis Intervention Study, Fajara, The Gambia; Saint Louis University, United States of America

## Abstract

**Background:**

Hepatocellular Carcinoma (HCC) is a common malignancy worldwide with a high burden in West Africa. Male to female ratios show consistent bias toward males, the biological bases and variations of which are not well understood. We have used data from the Gambian National Cancer Registry to compare trends in incidence of HCC in both genders.

**Methods and Findings:**

Two periods were compared, 1988–1997 (early) and 1998–2006 (recent). In addition, the regression program joinpoint was used to assess trends over 19 years. Differences with self-reported ethnicity were assessed for the recent period using population data from 2003 census. Male to female ratio showed a significant decrease between the two periods from 3.28∶1 (95% CI, [2.93–3.65]) to 2.2∶1 (95% CI, [1.99–2.43]). Although rates in males were relatively stable (38.36 and 32.84 for, respectively, early and recent periods), they increased from 11.71 to 14.9 in females with a significant Annual Percentage Change of 3.01 [0.3–5.8] over 19 years and an increase in number of cases of 80.28% (compared to 26% in males). Significant variations in HCC risk, but not in gender ratio were observed in relation with ethnicity.

**Conclusion:**

This analysis of the only national, population-based cancer registry in West Africa shows a significant increase in HCC in females over recent years. This increase may be the consequence of major changes in lifestyle or viral risk factors, in particular obesity and hepatitis C, which have both been documented to increase in West Africa during recent years.

## Introduction

The Gambia is the smallest country of continental Africa and its population comprises diverse different ethnic groups also present in other countries of West Africa. It is the only African country with population-based, nationwide cancer registration (The Gambia National Cancer Registry). The Gambia National Cancer Registry was established in 1986 in the framework of the Gambia Hepatitis Intervention Study (GHIS) to provide data on the incidence of all cancers, with particular emphasis on liver cancer, the end-point of GHIS. The GHIS is a collaborative project between the Government of The Gambia, the International Agency for Research on Cancer, and the Medical Research Council of the United Kingdom. Its aim is to evaluate the protective effectiveness of hepatitis B vaccination in childhood against chronic liver disease, namely cirrhosis and primary liver cancer, in adulthood [1]. Worldwide, liver cancer is the sixth most common cancer estimated for the year 2002, with over 80% of all cases occurring in low resource and emerging countries [2]. The male to female ratio shows a consistent bias toward males, varying between 4∶1 and 2∶1, depending upon geographic area [3]. Although this male to female discrepancy is not completely understood, several explanations have been proposed such as the impact of alcohol consumption, cigarette smoking, higher levels of hepatic iron, higher risk of infection by Hepatitis B Virus or Hepatitis C Virus in men as compared to women and differential effects of androgens on the proliferation of hepatocellular carcinoma cells [3], [4]. In West Africa and in The Gambia in particular, liver cancer (hepatocellular carcinoma) is by far the most common cancer among men and the second among women (after cervix cancer) with a male to female ratio of about 3.18∶1 [5], [6]. In contrast to western countries, alcohol consumption and cigarette smoking are negligible risk factors in The Gambia [7]–[9]. Well-known risk factors for hepatocellular carcinoma in this region are exposure to dietary aflatoxin combined to chronic Hepatitis B Virus and/or Hepatitis C Virus infection. Data from a case-control study indicate that the prevalence of chronic hepatitis carriage in healthy adults in The Gambia is 15.6% for Hepatitis B Virus and 2.7% for Hepatitis C Virus [10]. In this study, we have used data from the Gambia National Cancer Registry to assess the variations in liver cancer incidence in relation with age, gender, and ethnicity, three variables registered in the Gambia National Cancer Registry. We have performed this analysis over two periods, 1988–1997 and 1998–2006.

## Results

### Liver cancer incidence

Over the past 19 years (1988–2006) the Gambia National Cancer Registry recorded a total of 2975 cases of liver cancer, including 2179 in males and 796 in females. Incidence data over the first 10 years of registration (1988–1997) have been previously reported [6]. Recent data (1998–2006) confirmed these earlier observations. Overall, cancer incidences were very low in both genders. Liver cancer was the most frequent cancer, representing 62% of all cancer cases among men recorded in the Gambia National Cancer Registry, with an Age standardized rate (ASR) of 32.84 (95% CI, [30.97–34.70]) per 10^5^ person-years. In women, it represented 28% of all cancers, with an ASR of 14.90 (95% CI, [13.62–16.17]) per 10^5^ person-years, and ranking second most common cancer after cervix cancer (15.45 (95% CI, [14.18–16.66]) per 10^5^ person-years) (Table 1). The main basis for diagnosis was clinical observation. Only a minority of cases was assessed by ultrasonography (40% of cases for the recent period) and levels of alpha foetoprotein (>100 ng/ml; measured on average in 33% for women and 42% for men) (Table S1). Histopathological confirmation of diagnosis was not available due to ethical and clinical restrictions on obtaining liver biopsies from patients with advanced liver cancer. HBsAg status was known for less than 30% of the patients among which 54.2% and 36.6% were positive for HBsAg among men and women respectively. The mean age at diagnosis was 46 years for women and 45 years for men.

**Table 1:** Most common cancers in The Gambia for the period 1998–2006.

	Men			Women		
Site	Total Number of cases	ASR (w) per 100000	% of all cancers	Total number	ASR (w) per 100000	% of all cancers
Oesophagus	17	0,55	0,87	14	0,51	0,76
Stomach	48	1,58	2,45	17	0,59	0,92
Colorectal, Anus	40	1,17	2,04	28	0,93	1,51
Liver	1215	32,84	61,99	512	14,90	27,69
Pancreas	22	0,73	1,12	13	0,51	0,70
Trachea, bronchus and lung	71	2,46	3,62	10	0,39	0,54
Kaposi sarcoma	21	0,51	1,07	9	0,20	0,49
Soft tissues	25	0,60	1,28	32	0,94	1,73
Breast	11	0,35	0,56	215	5,82	11,63
Cervix	-	-	-	545	15,45	29,48
Corpus uteri	-	-	-	31	0,92	1,68
Ovary	-	-	-	36	0,97	1,95
Prostate	95	3,46	4,85	-	-	-
Bladder	29	0,91	1,48	8	0,21	0,43
Thyroid	11	0,25	0,56	36	0,90	1,95
Non-Hodgkin lymphoma	88	1,42	4,49	67	1,29	3,62

### Liver cancer trends

**Figure 1:**
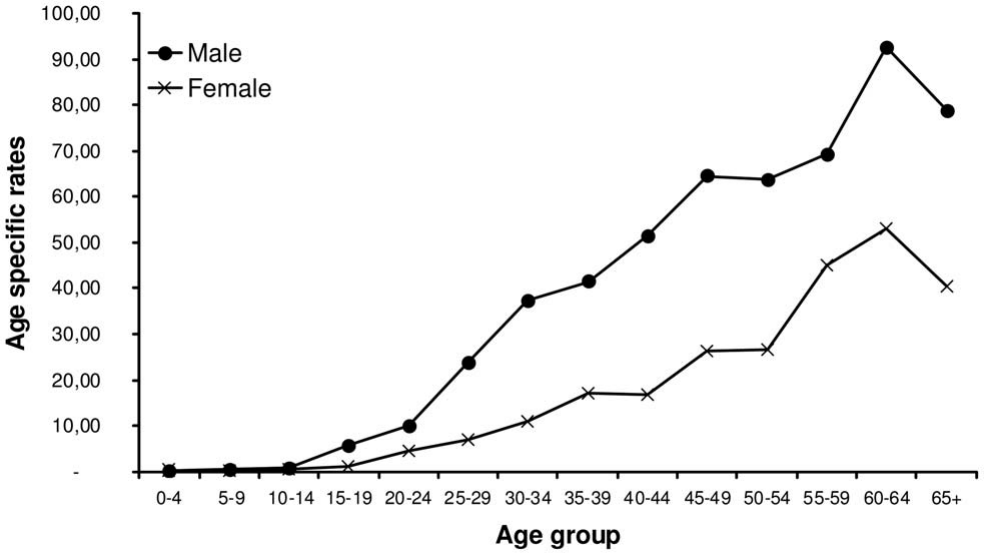
Liver cancer incidence rates per 100000 Gambians by 5-years age groups. Data from the Gambia National Cancer Registry, 1998–2006.

The liver cancer burden curves by age group showed a progressive increase with age in both men and women. Nevertheless, compared to men, the increase in incidence rates was slower and more progressive among women, who showed rates on average 2 fold lower compared to men (Figure 1). Comparing the early period (1988–1997) to the recent one (1998–2006), the male to female ratio showed a decrease of 33%, from 3.28∶1 (95% CI, [2.93–3.65]) to 2.2∶1 (95% CI, [1.99–2.43]) respectively. Indeed, from the period 1988–1997 to 1998–2006, the ASR among men has slightly decreased from 38.36 to 32.84 (ASR ratio 0.86 95% CI, [0.79–0.93]), while among women, it has increased from 11.71 to 14.90 for the early and recent period respectively (ASR ratio 1.27 95% CI, [1.12–1.45]) (Table 2).

**Table 2:** Comparison of the ASRs evolution by gender for the period 1988–1997 and 1998–2006.

	1988–1997	1998–2006	Evolution
	Cases	ASRs	95% Cis	Cases	ASRs	95% Cis	Cases	ASR Ratio	95% Cis
Male	964	38.36	36.16–40.56	1215	32.84	30.97–34.71	26.04%	0.86	0.79–0.93
Female	284	11.71	10.57–12.85	512	14.9	13.63–16.17	80.28%	1.27	1.12–1.45
M/F Ratio		3.28	2.93–3.65		2.2	1.99–2.43	−33%^1^		

**^1^**Male to female (M/F) ratio evolution

**Figure 2:**
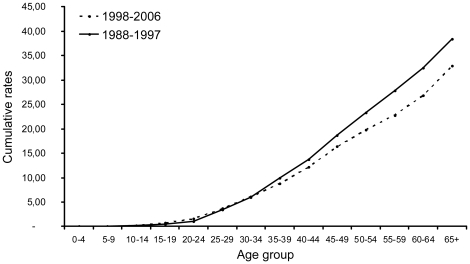
Male comparative liver cancer trends over two periods of 10 (1988–1997) and 9 (1998–2006) years, by age group.

**Figure 3:**
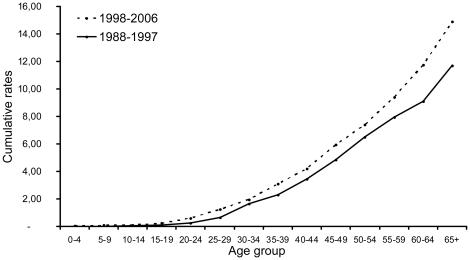
Female comparative liver cancer trends over two periods of 10 (1988–1997) and 9 (1998–2006) years, by age group.

The cumulative curves by age group for men and women (Figures 2, 3), comparing the rate by age group for the two periods (recent to early) showed a small decrease of rate among men in particular after the age group 30–34 while among women, there was a clear increase of rate in all age groups. When comparing each year of the two periods (Figures S1, S2), the incidence rates for men were relatively stable with a decrease in years 2000–2005 and a modest increase in 2006. In contrast, the cumulative rates for women showed an increase in rates for all years of the recent period. However, the lowest increase was observed for years 2001–2003, concordant with the decrease observed in men. This effect might reflect a change in liver cancer detection strategies that resulted in under ascertainment of liver cancers, principally in older age groups, in the years 2001–2005, and which was corrected in more recent years. This effect is obvious in males, but is less visible in females, in whom it is largely compensated by an increase in all age groups. Using joinpoint regression program over the past 19 years (1988–2006), the slope for men presented a non-significant annual percentage change (APC) of 0.02 [−1.8; 1.9] (Pvalue  = 0.980). The apparent discrepancy between this joinpoint analysis and the modest decrease shown in Table 2 and Figure 2a may be explained by the rather wide dispersion of annual data points. In contrast, among women, the joinpoint regression shows a significant APC of 3.01 [0.3; 5.8] (P value  = 0.028) (Figures S3, S4).

### Differences according to ethnic groups

There were significant differences among ethnic groups in the relative risk of liver cancer for the period 1998–2006. Considering the Mandinka (most numerous group) as reference, the Fula and Wollof had respectively a significantly higher risk of 2.15 (95% CI, [1.83–2.53]) and 1.78 (95% CI, [1.50–2.10]) in men. In contrast, the Serrahuleh had a lower significant risk (0.61, 95% CI, [0.44–0.84]). Among women, the Fula and Wollof had a significant higher risk of 1.94 (95% CI, [1.51–2.48]) and 1.80 (95% CI, [1.38–2.35]) as compared to the Mandinka (Table 3). After adjustment for district of residence, the Wollof presented the highest significant risk in both genders (1.55 (95% CI, [1.31–1.84), and 1.37 (95% CI, [1.05–1.79) in male and female respectively, as compared to Mandinka. In Fula, a significantly increased risk of 1.40 (95% CI, [1.20–1.65]) was observed in males, whereas it was only slightly and non-significantly increased in females (1.22 (95% CI, [0.95–1.79)) (Table 3). Table S2 shows unadjusted rate ratios in relation with division of residence and the proportion of different ethnic groups in the population of each division, respectively.

**Table 3:** Male and female liver cancer by ethnic groups in The Gambia 1998–2006.

Site	Ethnic groups	% in the population	Crude		Adjusted to district of residence	
			Rate Ratio	CI 95%	Rate Ratio	CI 95%
Male liver cancer						
	Mandinka	42	(reference category)	-	(reference category)	-
	Fula	18	2.15	1.83–2.53	1.40	1.20–1.65
	Wollof	16	1.78	1.50–2.10	1.55	1.31–1.84
	Jola	10	1.10	0.87–1.40	0.91	0.71–1.15
	Serrahuleh	9	0.61	0.44–0.84	0.73	0.53–1.01
Female liver cancer						
	Mandinka	42	(reference category)	-	(reference category)	-
	Fula	18	1.94	1.51–2.48	1.22	0.95–1.56
	Wollof	16	1.80	1.38–2.35	1.37	1.05–1.79
	Jola	10	1.29	0.90–1.86	1.19	0.83–1.73
	Serrahuleh	9	1.20	0.80–1.81	0.73	0.49–1.11

## Discussion

Despite the fact that liver cancer is the main form of cancer in West Africa, there is little information available on its variations with age, time, gender and ethnicity. The Gambian National Cancer Registry has collected structured, nationwide information on liver cancer since 1986. One of the limitations of the data collected by the registry is the paucity of diagnostic information, with only about 40% of the cases assessed by liver ultrasonography. However, the diagnosis of liver cancer is unlikely to be strongly biased by other, space-filling liver lesions. A recent study of 323 clinically assessed cases from The Gambia showed that over 90% of the cases presented with very advanced liver cancer, 45% of them having tumors with a diameter over 10 cm. More than two-third had multiple lesions and 41% had lesions in both liver lobes. The main clinical features were hepatomegaly (92%), abdominal pain (94%) and weight loss (94%). This triad was present in 74% of the patients and the median duration of reported symptoms prior to HCC diagnosis was 8 weeks [11]. Furthermore, only 67% of the cases showed evidence of cirrhosis at the time of HCC diagnosis. A morphological study of 35 liver biopsies obtained in the course of a case-control study confirmed HCC in 29 (83.3%) of patients having been diagnosed only on the basis of clinical criteria. The 6 excluded cases were 1 liver hemangioma and 5 liver metastases of unspecified primary tumors. The specificity increased to 95% using the combination of clinical assessment, a-foetoprotein levels and ultrasonography [12]. Of note, cases of liver metastases of identified primary tumors were excluded from the present analysis.

Analysis of liver cancer incidence data over two time periods of registration (1988–1997 and 1998–2006) has allowed us to draw two main observations. First, between the two periods, there has been a decrease in male to female ratio (from 3.28 to 2.20, −33%), and this decrease is essentially attributable to an increase in females in all age groups. A modest decrease between the two registration periods is also noted in men aged over 30 years. However, in contrast with women, the decrease in men is no longer apparent in a regression analysis that takes into account each year's data over the two periods. Thus, it rates in men should be considered as relatively stable over the period covered by this study. In our analyses, there was no apparent decrease in liver cancer rates in young subjects despite the initiation of a vaccination program in 1986 (GHIS). It should be noted that the introduction of vaccination in The Gambia was performed in a stepwise manner during years 1986 to 1990, so that only half of the subjects born during that period actually received HB vaccine. Thus, the results presented here cannot be interpreted as revealing an absence of effect of vaccination in the younger age group (15–19 years). A recent review of the GHIS has shown that a significant effect of vaccination may not be measurable before 2017 [1].

A significant decrease over time in male to female ratio for liver cancer has been observed in the Uganda (Kyadondo) cancer registry. In this area, the ASR of liver cancer for men is 8.7 per 10^5^ person-years and 5.8 for women (ranking 4^th^ and 8^th^ among men and women respectively) [13]. Comparison between the periods 1960–1980 and 1991–2005 showed that liver cancer incidence (ASR) was quite stable or even slightly decreased among men, but increased by over 50% among women, with a change in male to female ratio from 2.32 to 1.30 [14]. Thus, our observations in The Gambia may correspond to a phenomenon which may also occur in other Sub-Saharan African countries.

Several explanations may be proposed. First, it is possible that female liver cancer has been systematically under-detected and underestimated over earlier cancer registration periods, and that the increase in the recent period (1998–2006) may primarily reflect improved registration in women. It should be noted, however, that no comparable change has been observed for other female cancers (breast and cervix cancer in particular [5]), hence it is unclear why there should be a registration bias only for liver cancer. A second explanation may be changes in the prevalence of liver cancer risk factors and in their contribution to liver cancer burden in each gender. Three main factors may be considered: chronic infection by hepatitis B virus (Hepatitis B Virus), infection by hepatitis C virus (Hepatitis C Virus) and metabolic conditions associated with obesity and/or diabetes type 2.

Case control studies have shown that much of the imbalance in liver cancer incidence between men and women may be attributable to Hepatitis B Virus. In a study performed between 1997 and 2001, [10] the prevalence of chronic Hepatitis B Virus carriage was of 18% in men and 10% in women (p = 0.05) with a risk of liver cancer 2.6 (95% CI, [1.5–4.6]) fold higher in men than in women. In contrast, the prevalence of chronic Hepatitis C Virus infection was similar in both genders (2.6% in males and 3.6% in females, p = 0.57). Among liver cancer patients, 32% of women were Hepatitis C Virus positive, as compared to 16% of men (p = 0.03). Thus, the decrease in male to female ratio may be suggestive of an increasing impact of Hepatitis C Virus as cause of liver cancer. The possibility that Hepatitis C Virus may contribute to the increasing burden of female liver cancer deserves further investigation.

The other factor that may account for the observed increase in incidence of liver cancer in female is an increase in the prevalence of metabolic disorders associated with obesity, physical inactivity and/or diabetes type 2. Recent meta-analyses have found that in West Africa, women were more likely to be obese than men (odds ratio 3.16 95% CI, [2.51–3.98] and 4.79 (95% CI, [3.30–6.95]) in urban and rural areas, respectively [15]. Time trend analyses indicated that the prevalence of obesity in urban West Africa had more than doubled (114%) over 15 years, accounted for almost entirely in women [15]. Although data are limited, differences in overweight and obesity by gender and age have been observed in The Gambia. Among subjects aged 35–50 years (the age group with high rates of liver cancer), women were more likely to be overweight or obese as compared to men (34% versus 6%, and 50% versus 6%, respectively) [16]. Cumulative time trends analyses suggested an increase in the prevalence of diabetes among adults in urban West Africa, from approximately 3% to 4% in the past 10 years, with similar prevalence and trends in males and females [17]. A recent meta-analysis of 11 cohort studies including cohorts from Western Europe, US and Korea has shown a substantial association between excess bodyweight and increased risk of liver cancer. In this meta-analysis, compared with persons of normal weight, the relative risk of liver cancer was 1.89 (95% CI: 1.51–2.36) for those who were obese [18]. Although there is currently no data on West African populations, it is plausible that overweight may also contribute to the increased burden of liver cancer in Gambian females. These associations need to be further tested in case-control and cohort studies.

Our results report a significant association between the risk of liver cancer and ethnicity. A recent genome-wide association analysis of malaria in The Gambia demonstrated that self-reported ethnicity correlates with genetically defined subpopulations [19], [20]. Our analyses show that Fula (18% of the population) and Wollof had a significantly higher risk of liver cancer than Mandinka (42% of the population). This association was previously reported in a case-control study in The Gambia [10]. Two studies conducted on Gambian children reported that blood levels of aflatoxin-albumin adducts were slightly higher in Fula and Wollof than Mandinka [21], [22]; suggesting that these groups may have a higher sensitivity to aflatoxin due to genetic polymorphisms in enzymes involved in aflatoxin metabolism, and detoxification. In a case-control study, Fula were found to have a significantly higher prevalence than Mandinka in the Gly399 allele of *XRCC1*, an enzyme involved in excision repair of aflatoxin-DNA adducts [23]. These observations suggest that the higher risk observed in selected groups may be due to genetic susceptibility, although differences in lifestyle may also play an important role, in particular among rural populations.

In conclusion, our study based on almost two decades of population-based cancer registration in The Gambia identifies a significant trend in increase of hepatocellular carcinoma among females, with a significant reduction of the male to female ratio. This observation may be the consequence of changes in lifestyle or viral risk factors and suggest that recent increase in the prevalence of obesity among women deserves further attention in hepatocellular carcinoma prevention strategies.

## Methods

### Liver cancer case definition

The case definition for HepatoCellular Carcinoma (HCC) is based on a combination of a compatible clinical assessment, a-foetoprotein testing (levels ≥100 ng/ml) and positive ultrasonography findings. This definition was assessed against histopathology using a limited number of good quality biopsies obtained in the course of a case-control study (Szymanska et al., 2003) and showed a specificity of over 95% for HCC. Clinical assessment alone showed a specificity over 80%.

### Cancer incidence data

Methods and procedures of data collection of the Gambia National Cancer Registry have been described previously [6]. Data are centralized at the GHIS office in Fajara, verified, and managed using the CanReg 4.0 software [24] (http://www.iacr.com.fr/) and ICD-O-3 (international classification of disease for oncology third edition) for cancer coding. Computerized and verified data in the Gambia National Cancer Registry was available until the years 2006. The first two years of cancer registration (1986 and 1987) were excluded since it was clear that case finding was incomplete for these two years. The rest of the available years of cancer registration activity (1988–2006) were arbitrarily divided into two periods of 9 and 10 years, respectively (1988–1997 and 1998–2006). Incidence rates for the more recent period (1998 to 2006) were assessed by gender, age group, years and ethnicity using the person-years estimates from three nationwide censuses carried out in 1983, 1993 and 2003. Population age structure of these censuses were linearly interpolated for period from 1988 to 1992, 1994 to 2002, and extrapolated for period from 2004 to 2006. Because of the lack of information concerning ethnicity in the censuses 1983 and 1993, ethnic variation analysis was restricted to the recent period (1998–2006).

### Information on ethnicity

The Gambia population includes about a dozen distinct ethnic groups, all of which are also present in other countries of West Africa [25]. Information on ethnicity was self-reported at diagnosis (each subject identifying him/herself based on the language and culture of biological parents) or was obtained during a structured interview by Gambia National Cancer Registry clerks who are fluent in the language of each major ethnic group. This information was complete at 87% for men and 86% for women. Although the variable “ethnicity” captures different sources of variations including lifestyle, cultural, geographic and possible genetic factors, a recent genome-wide association analysis of malaria in the Gambia demonstrated that self-reported ethnicity correlates with genetically defined subpopulations [26].

Population distributions for the 5 main ethnic groups was based on the 2003 census in which the Mandinka, Fula, Wollof, Jola and Serrahuleh represented 42%, 18%, 16%, 10% and 9% of the total population respectively (http://www.accessgambia.com/information/people-tribes.html, https://www.cia.gov/library/publications/the-world-factbook/geos/ga.html#People). In the absence of detailed demographic information, it was assumed that age and sex population structure in each ethnic group was identical to that of the whole population. Of note, in the 2003 census, ethnicity was self-reported.

### Statistical analysis

Age standardized incidence rates were obtained by adjusting the incidence rate to the world population by a 5-year age group as described by Boyle and Parkin in the book “enregistrement des cancers principes et methodes” [27]. Joinpoint regression program version 3.3 (http://srab.cancer.gov/joinpoint/) was used to assess trends of liver cancer over 19 years (1988–2006). To investigate ethnic variations in incidence rates (1998–2006 period only), Poisson regression models were fitted with number of cases as the response variable and categorical 5-year age group and ethnic group as explanatory variables. Population size was taken into account in modelling by using it as an offset term in Poisson models. All analyses were carried using R software (3). Ethnic groups have historically distinct geographic distribution in the country as shown in Table S2. Since access to diagnosis may differ between urban and rural areas, residence was considered as a confounding factor. Therefore, incidence rate was adjusted to the place of residence by stratifying the different ethnic group by place of residence grouped in district. The adjusted rate ratio was then obtained by using Mantel and Haenszel's method as described in the text book “Epidémiologie du cancer principes et méthodes chapter 14” [28]. The Mandinka, who represent the most numerous ethnic group, was used as the reference group.

## Supporting Information

Table S1 Liver cancer detail information in The Gambia (data of the cancer registry).
(DOC)

Table S2 Liver cancer crude rates by Gambia Divisions (The Gambia 1998–2006).
(DOC)

Figure S1 Comparative liver cancer trends in males over two periods of 10 (1988–1997) and 9 (1998–2006) years, by period year.
(DOC)

Figure S2 Comparative liver cancer trends in females over two periods of 10 (1988–1997) and 9 (1998–2006) years, by period year.
(DOC)

Figure S3 Males liver cancer trends in the Gambia 1988–2006 0 joinpoint.
(DOC)

Figure S4 Females liver cancer trends in the Gambia 1988–2006 0 joinpoint.
(DOC)
