# Supplementary material for: Increase in Female Liver Cancer in The Gambia, West Africa: Evidence from 19 Years of Population-Based Cancer Registration (1988–2006)
Source: PLoS One. 2011 Apr 7;6(4):e18415. doi: 10.1371/journal.pone.0018415 (PMC3072390; doi:10.1371/journal.pone.0018415)
Supplement: Table S1 — Liver cancer detail information in The Gambia (data of the cancer registry). (DOC) [file pone.0018415.s001.doc]

Table 1: Liver cancer detail information in The Gambia (data of the cancer registry)
