# Supplementary material for: Increase in Female Liver Cancer in The Gambia, West Africa: Evidence from 19 Years of Population-Based Cancer Registration (1988–2006)
Source: PLoS One. 2011 Apr 7;6(4):e18415. doi: 10.1371/journal.pone.0018415 (PMC3072390; doi:10.1371/journal.pone.0018415)
Supplement: Table S2 — Liver cancer crude rates by Gambia Divisions (The Gambia 1998–2006). (DOC) [file pone.0018415.s002.doc]

Table 2: Liver cancer crude rates by Gambia Divisions (The Gambia 1998 - 2006)

| Male | | | |  | Female | | |  | Proportion of ethnic groups by district**^2^** | | | | |
| --- | --- | --- | --- | --- | --- | --- | --- | --- | --- | --- | --- | --- | --- |
| Gambia  Divisions | Crude rate  per 10^5^ | Rate ratio | CI 95% |  | Crude rate per 10^5^ | Rate ratio | CI 95% |  | Mandinka | Fula | Wollof | Jola | Serrahuleh |
|  |  |  |  |  |  |  |  |  |  |  |  |  |  |
| Kerewan  Banjul | 19,12  19,05 | 1,34  1,33 | 0.82-2.17  0.82-2.16 |  | 7,50  8,74 | 1,01  1,18 | 0.71-1.44  0.87-1.59 |  | 36%  30% | 20%  15% | 30%  22% | 1%  13% | 1%  5% |
| Brikama | 14,86 | 1,04 | 0.83-1.30 |  | 5,18 | 0,70 | 0.50-0.96 |  | 41% | 16% | 5% | 23% | 1% |
| Mansa konko | 18,84 | 1,32 | 0.81-2.14 |  | 4,51 | 0.61 | 0.35-1.07 |  | 62% | 27% | 3% | 2% | 3% |
| Central River | 24,84 | 1,73 | 1.12-2.68 |  | 11,39 | 1,53 | 1.11-2.11 |  | 26% | 40% | 23% | 1% | 4% |
| Basse | 14,32 | (reference category) | |  | 7,43 | (reference category) | |  | 31% | 27% | 0,40% | 0,30% | 39% |

**^2^** <http://www.columbia.edu/_msj42/pdfs/Chapter6_Nationality_enthnicity.pdf>.

Ethnic distribution in percentage of the different districts of The Gambia.
