# Supplementary material for: Increase in Female Liver Cancer in The Gambia, West Africa: Evidence from 19 Years of Population-Based Cancer Registration (1988–2006)
Source: PLoS One. 2011 Apr 7;6(4):e18415. doi: 10.1371/journal.pone.0018415 (PMC3072390; doi:10.1371/journal.pone.0018415)
Supplement: Figure S2 — Comparative liver cancer trends in females over two periods of 10 (1988–1997) and 9 (1998–2006) years, by period year. (DOC) [file pone.0018415.s004.doc]

**Figure 2:** Comparative liver cancer trends in females over two periods of 10 (1988-1997) and 9 (1998-2006) years, by period year.
