# Supplementary figures and images for: Increase in Female Liver Cancer in The Gambia, West Africa: Evidence from 19 Years of Population-Based Cancer Registration (1988–2006)
Source: PLoS One. 2011 Apr 7;6(4):e18415. doi: 10.1371/journal.pone.0018415 (PMC3072390; doi:10.1371/journal.pone.0018415)

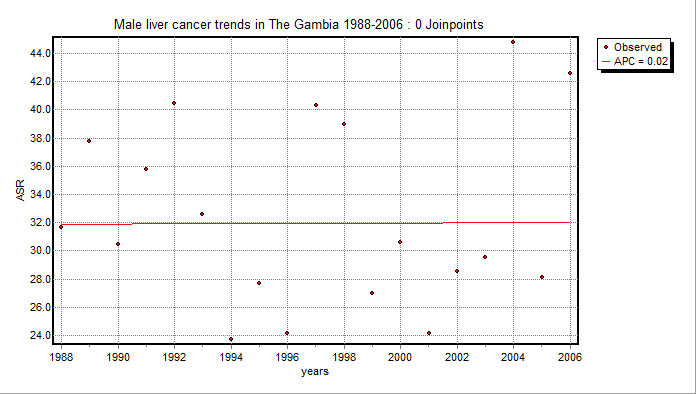


* Significant Pvalue < 0.05

**Figure 3:** Males liver cancer trends in the Gambia 1988-2006 0 joinpoint.

Supplement: Figure S3 — Males liver cancer trends in the Gambia 1988–2006 0 joinpoint. (DOC) [file pone.0018415.s005.doc]

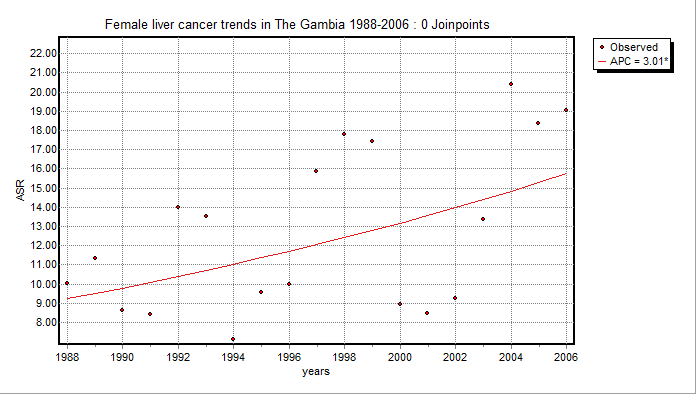


* Significant Pvalue < 0.05

**Figure 4:** Females liver cancer trends in the Gambia 1988-2006 0 joinpoint.

Supplement: Figure S4 — Females liver cancer trends in the Gambia 1988–2006 0 joinpoint. (DOC) [file pone.0018415.s006.doc]
